# (1*R*,3*S*)-Methyl 3-[(*S*)-2-(hydroxy­diphenyl­meth­yl)pyrrolidin-1-ylmeth­yl]-2,2-dimethyl­cyclo­propane­carboxyl­ate

**DOI:** 10.1107/S1600536809035296

**Published:** 2009-09-16

**Authors:** Bo Wang, Nengsheng Ye, Zhiyuan Li, Jiangchun Zhong, Shicong Hou

**Affiliations:** a Department of Chemistry, Capital Normal University, 100089 Beijing, People’s Republic of China; b Department of Applied Chemistry, China Agriculture University, 100193 Beijing, People’s Republic of China

## Abstract

The asymmetric unit of the title compound, C_25_H_31_NO_3_, prepared from (−)-1*R*-*cis*-caronaldehyde, contains three independent mol­ecules with similar conformations. The hydr­oxy groups are involved in intra­molecular O—H⋯N hydrogen bonds. The crystal packing exhibits weak inter­molecular O—H⋯O and C—H⋯O hydrogen bonds.

## Related literature

For details of the preparation of an analogous compound, see: Bakshi *et al.* (1989 ▶); Mattson *et al.* (1990 ▶). For a related structure, see: Na & Wang (2009 ▶).
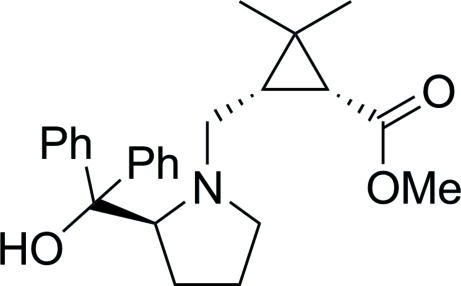


## Experimental

### 

#### Crystal data

C_25_H_31_NO_3_
*M* *_r_* = 393.51
Monoclinic, 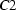

*a* = 34.736 (7) Å
*b* = 17.729 (4) Å
*c* = 11.013 (2) Å
β = 97.62 (3)°
*V* = 6722 (2) Å^3^
*Z* = 12
Mo *K*α radiation
μ = 0.08 mm^−1^
*T* = 173 K
0.50 × 0.33 × 0.29 mm

#### Data collection

Rigaku Saturn724+ CCD diffractometer
Absorption correction: numerical *ABSCOR* (Higashi, 1995 ▶) *T*_min_ = 0.963, *T*_max_ = 0.978
41275 measured reflections
7960 independent reflections
7677 reflections with *I* > 2σ(*I*)
*R*_int_ = 0.050

#### Refinement

*R*[*F* ^2^ > 2σ(*F* ^2^)] = 0.059
*wR*(*F* ^2^) = 0.145
*S* = 1.17
7960 reflections
784 parameters
1 restraint
H-atom parameters constrained
Δρ_max_ = 0.41 e Å^−3^
Δρ_min_ = −0.24 e Å^−3^

### 

Data collection: *CrystalClear* (Rigaku, 2005 ▶); cell refinement: *CrystalClear*; data reduction: *CrystalClear*; program(s) used to solve structure: *SHELXS97* (Sheldrick, 2008 ▶); program(s) used to refine structure: *SHELXL97* (Sheldrick, 2008 ▶); molecular graphics: *XP* in *SHELXTL* (Sheldrick, 2008 ▶); software used to prepare material for publication: *SHELXL97*.

## Supplementary Material

Crystal structure: contains datablocks I, global. DOI: 10.1107/S1600536809035296/cv2590sup1.cif

Structure factors: contains datablocks I. DOI: 10.1107/S1600536809035296/cv2590Isup2.hkl

Additional supplementary materials:  crystallographic information; 3D view; checkCIF report

## Figures and Tables

**Table 1:** Hydrogen-bond geometry (Å, °)

*D*—H⋯*A*	*D*—H	H⋯*A*	*D*⋯*A*	*D*—H⋯*A*
O1*B*—H1*BA*⋯N1*B*	0.86	2.08	2.682 (4)	127
O1*A*—H1*A*⋯N1*A*	0.84	2.30	2.650 (4)	105
O1*C*—H1*C*⋯N1*C*	0.84	2.31	2.679 (4)	107
C14*C*—H14*B*⋯O1*B*^i^	1.00	2.51	3.319 (4)	138
C19*B*—H19*A*⋯O2*C*^ii^	1.00	2.37	3.321 (4)	158
O1*A*—H1*A*⋯O2*B*^iii^	0.84	2.55	3.236 (4)	140

Symmetry codes: (i) 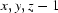; (ii) 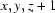; (iii) 
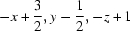
.

**Fig. 1.**
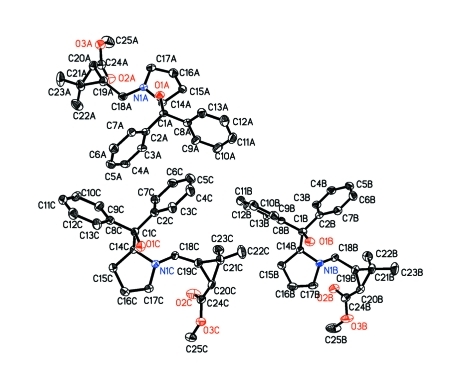
Three independent molecules of (I), with atomic labels and 30% probability displacement ellipsoids. H atoms omitted for clarity.

## supplementary crystallographic information

### Comment

In continuation of our study of new (-)-1*R*-*cis*-caronaldehyde derivatives (Na &Wang, 2009), herewith we present the crystal structure of the title compound, (I).

The asymmetric unit of (I) contains three independent molecules (Fig. 1). The hydroxy groups are involved in intramolecular O—H···N hydrogen bonds (Table 1). The crystal packing exhibits weak intermolecular O—H···O and C—H···O hydrogen bonds (Table 1).

### Experimental

(1*R*,5*S*)-4-Hydroxy-6,6-dimethyl-3-oxa-bicyclo[3.1.0]-hexan-2-one1 (42.6 g, 300 mmol) was dissolved in a mixture of 250 mL of MeOH containing *p*-toluene sulfonic acid (10 g, 58 mmol) and refluxed for 5 hr. When reaction mixture cooled, sodium acetate (10 g) was added to mixture and MeOH was removed underreduced pressure. The residue was dissolved in *N*-hexane (250 mL), washed with water (3×100 mL) and freed of solvent. The residuewas taken up in 0.5% aq oxalic acid (200 mL) and stirred at room temperature for 10 h and extracted with Et_2_O (3×100 mL). The combined organic layer was dried over anhydrous Na_2_SO_4_, and the solvent was removed under reduced pressure, yield 35 g coloress oily. The crude product was not purified for next step (Bakshi *et al.*, 1989). A mixture of the previous product (20 mmol), diphenyl-pyrrolidinemethanol (20 mmol), and titanium (IV) is opropoxide (7.5 mL,12.5 mmol) was stirred at room temperature in a 100 mL flask under a drying tube. After 1 h, the viscous solution was diluted with absolute methanol (20 mL). The solution became bright yellow. Sodium cyanoborohydride (0.84 g, 13.4 mmol) was added, and the solution was stirred for 20 h. Water (4 mL) was added with stirring,and the resulting inorganic precipitate was filtered and washed with methanol.The filtrate was then concentrated *in vacuo*. The crude product was dissolved in ether, filtered to remove the remaining inorganic solids, and concentrated in vacuo. The product was then purified by flash chromatography. Colourless solid, m.p. 393 K; [α]_20_^D^ =-5.74 (*c* 0.01, CHCl_3_) (Mattson *et al.*, 1990).

### Refinement

All H atoms were positioned geometrically [C—H 0.95-1.00 Å; O—H 0.84-0.86 Å], and refined as riding, with *U*_iso_ (H) = 1.2-1.5 Ueq of the parent atom. In the absence of anomolous scatterers, 7396 Friedel pairs were merged before the final refinement.

### Crystal data

**Table:**

C_25_H_31_NO_3_	*F*(000) = 2544
*M**_r_* = 393.51	*D*_x_ = 1.166 Mg m^−^^3^
Monoclinic, *C*2	Mo *K*α radiation, λ = 0.71073 Å
*a* = 34.736 (7) Å	Cell parameters from 768 reflections
*b* = 17.729 (4) Å	θ = 2.2–27.5°
*c* = 11.013 (2) Å	µ = 0.08 mm^−^^1^
β = 97.62 (3)°	*T* = 173 K
*V* = 6722 (2) Å^3^	Strip, colourless
*Z* = 12	0.50 × 0.33 × 0.29 mm

### Data collection

**Table:**

Rigaku Saturn724+ CCD diffractometer	7960 independent reflections
Radiation source: sealed tube	7677 reflections with *I* > 2σ(*I*)
graphite	*R*_int_ = 0.050
ω scans at fixed χ = 45°	θ_max_ = 27.5°, θ_min_ = 2.3°
Absorption correction: numerical *ABSCOR* (Higashi, 1995)	*h* = −45→45
*T*_min_ = 0.963, *T*_max_ = 0.978	*k* = −23→23
41275 measured reflections	*l* = −14→14

### Refinement

**Table:**

Refinement on *F*^2^	Primary atom site location: structure-invariant direct methods
Least-squares matrix: full	Secondary atom site location: difference Fourier map
*R*[*F*^2^ > 2σ(*F*^2^)] = 0.059	Hydrogen site location: inferred from neighbouring sites
*wR*(*F*^2^) = 0.145	H-atom parameters constrained
*S* = 1.17	*w* = 1/[σ^2^(*F*_o_^2^) + (0.0632*P*)^2^ + 2.6224*P*] where *P* = (*F*_o_^2^ + 2*F*_c_^2^)/3
7960 reflections	(Δ/σ)_max_ = 0.001
784 parameters	Δρ_max_ = 0.41 e Å^−^^3^
1 restraint	Δρ_min_ = −0.24 e Å^−^^3^

### Special details

**Table:**

Geometry. All e.s.d.'s (except the e.s.d. in the dihedral angle between two l.s. planes) are estimated using the full covariance matrix. The cell e.s.d.'s are taken into account individually in the estimation of e.s.d.'s in distances, angles and torsion angles; correlations between e.s.d.'s in cell parameters are only used when they are defined by crystal symmetry. An approximate (isotropic) treatment of cell e.s.d.'s is used for estimating e.s.d.'s involving l.s. planes.
Refinement. Refinement of *F*^2^ against ALL reflections. The weighted *R*-factor *wR* and goodness of fit *S* are based on *F*^2^, conventional *R*-factors *R* are based on *F*, with *F* set to zero for negative *F*^2^. The threshold expression of *F*^2^ > σ(*F*^2^) is used only for calculating *R*-factors(gt) *etc*. and is not relevant to the choice of reflections for refinement. *R*-factors based on *F*^2^ are statistically about twice as large as those based on *F*, and *R*- factors based on ALL data will be even larger.

### Fractional atomic coordinates and isotropic or equivalent isotropic displacement parameters (Å^2^)

**Table:**

	*x*	*y*	*z*	*U*_iso_*/*U*_eq_	
O1A	0.61741 (6)	0.71980 (12)	0.0217 (2)	0.0404 (5)	
H1A	0.6068	0.7249	−0.0510	0.061*	
O1C	0.87641 (7)	0.55390 (12)	−0.0018 (2)	0.0445 (5)	
H1C	0.8875	0.5614	0.0697	0.067*	
O1B	0.87006 (7)	0.87326 (12)	0.9332 (2)	0.0475 (5)	
H1BA	0.8845	0.8878	0.9980	0.071*	
O2B	0.95265 (7)	1.17966 (15)	1.1925 (3)	0.0608 (7)	
O2A	0.55251 (9)	1.02781 (16)	−0.2743 (3)	0.0773 (9)	
O2C	0.95309 (8)	0.84700 (15)	0.3125 (4)	0.0753 (9)	
O3A	0.49276 (7)	0.98742 (15)	−0.3403 (3)	0.0585 (6)	
O3C	1.00025 (7)	0.76433 (16)	0.3560 (2)	0.0559 (6)	
O3B	1.01025 (8)	1.13285 (18)	1.2684 (4)	0.0783 (9)	
N1C	0.90248 (7)	0.68825 (17)	0.0848 (2)	0.0400 (5)	
N1A	0.58899 (7)	0.84984 (15)	−0.0687 (2)	0.0382 (5)	
N1B	0.90658 (7)	0.99525 (15)	1.0363 (2)	0.0396 (6)	
C1C	0.85125 (8)	0.61510 (16)	−0.0382 (3)	0.0342 (6)	
C1A	0.64291 (8)	0.78108 (16)	0.0535 (3)	0.0340 (6)	
C1B	0.84952 (8)	0.94225 (16)	0.9081 (3)	0.0364 (6)	
C2A	0.67399 (8)	0.78459 (17)	−0.0334 (3)	0.0360 (6)	
C2B	0.82002 (8)	0.95341 (16)	0.9997 (3)	0.0359 (6)	
C2C	0.82046 (9)	0.62294 (18)	0.0509 (3)	0.0370 (6)	
C3B	0.79781 (9)	1.01844 (19)	0.9986 (3)	0.0437 (7)	
H3BA	0.8006	1.0566	0.9398	0.052*	
C3C	0.81643 (10)	0.56596 (19)	0.1355 (3)	0.0457 (7)	
H3CA	0.8322	0.5221	0.1368	0.055*	
C3A	0.69694 (8)	0.84829 (19)	−0.0430 (3)	0.0429 (7)	
H3AA	0.6942	0.8907	0.0078	0.051*	
C4B	0.77159 (9)	1.0288 (2)	1.0822 (3)	0.0499 (8)	
H4BA	0.7565	1.0736	1.0796	0.060*	
C4A	0.72399 (9)	0.8506 (2)	−0.1263 (3)	0.0479 (8)	
H4AA	0.7395	0.8944	−0.1314	0.057*	
C4C	0.78966 (12)	0.5725 (2)	0.2177 (3)	0.0594 (10)	
H4CA	0.7874	0.5332	0.2749	0.071*	
C5A	0.72818 (9)	0.7893 (2)	−0.2012 (3)	0.0514 (8)	
H5AA	0.7461	0.7913	−0.2594	0.062*	
C5B	0.76748 (10)	0.9744 (2)	1.1686 (3)	0.0509 (8)	
H5BA	0.7497	0.9815	1.2262	0.061*	
C5C	0.76616 (11)	0.6356 (3)	0.2178 (3)	0.0587 (10)	
H5CA	0.7481	0.6401	0.2751	0.070*	
C6C	0.76942 (9)	0.6920 (2)	0.1327 (3)	0.0510 (8)	
H6CA	0.7532	0.7352	0.1304	0.061*	
C6A	0.70623 (10)	0.7253 (2)	−0.1910 (3)	0.0518 (8)	
H6AA	0.7094	0.6828	−0.2413	0.062*	
C6B	0.78927 (11)	0.9095 (2)	1.1707 (3)	0.0541 (9)	
H6BA	0.7865	0.8717	1.2300	0.065*	
C7B	0.81547 (9)	0.89896 (18)	1.0864 (3)	0.0435 (7)	
H7BA	0.8303	0.8539	1.0887	0.052*	
C7C	0.79635 (9)	0.6857 (2)	0.0507 (3)	0.0420 (6)	
H7CA	0.7984	0.7250	−0.0068	0.050*	
C7A	0.67956 (9)	0.72283 (18)	−0.1076 (3)	0.0406 (6)	
H7AA	0.6648	0.6782	−0.1009	0.049*	
C8B	0.82818 (8)	0.93710 (19)	0.7772 (3)	0.0410 (7)	
C8A	0.66136 (8)	0.76902 (17)	0.1854 (3)	0.0379 (6)	
C8C	0.83178 (9)	0.59895 (17)	−0.1678 (3)	0.0366 (6)	
C9C	0.80633 (10)	0.6500 (2)	−0.2317 (3)	0.0488 (8)	
H9CA	0.8003	0.6957	−0.1935	0.059*	
C9A	0.68768 (10)	0.8206 (2)	0.2450 (3)	0.0534 (9)	
H9AA	0.6954	0.8631	0.2014	0.064*	
C9B	0.82172 (10)	0.8679 (2)	0.7206 (3)	0.0523 (8)	
H9BA	0.8302	0.8230	0.7629	0.063*	
C10A	0.70284 (12)	0.8108 (3)	0.3671 (4)	0.0645 (11)	
H10A	0.7209	0.8462	0.4061	0.077*	
C10B	0.80270 (13)	0.8643 (3)	0.6005 (4)	0.0662 (12)	
H10B	0.7983	0.8166	0.5619	0.079*	
C10C	0.78971 (10)	0.6349 (2)	−0.3504 (3)	0.0559 (9)	
H10C	0.7722	0.6703	−0.3922	0.067*	
C11C	0.79803 (10)	0.5699 (2)	−0.4089 (3)	0.0540 (9)	
H11A	0.7867	0.5604	−0.4908	0.065*	
C11A	0.69168 (12)	0.7497 (3)	0.4321 (4)	0.0622 (10)	
H11B	0.7016	0.7435	0.5161	0.075*	
C11B	0.79036 (12)	0.9281 (3)	0.5377 (4)	0.0675 (12)	
H11C	0.7776	0.9249	0.4561	0.081*	
C12B	0.79658 (11)	0.9970 (3)	0.5934 (4)	0.0611 (10)	
H12A	0.7880	1.0417	0.5504	0.073*	
C12C	0.82314 (12)	0.5183 (2)	−0.3466 (3)	0.0551 (9)	
H12B	0.8290	0.4726	−0.3852	0.066*	
C12A	0.66611 (12)	0.6982 (2)	0.3742 (3)	0.0592 (9)	
H12C	0.6586	0.6557	0.4182	0.071*	
C13C	0.83986 (11)	0.53344 (19)	−0.2275 (3)	0.0472 (7)	
H13A	0.8573	0.4978	−0.1860	0.057*	
C13A	0.65107 (10)	0.70757 (19)	0.2516 (3)	0.0479 (7)	
H13B	0.6335	0.6712	0.2127	0.057*	
C13B	0.81537 (10)	1.0015 (2)	0.7127 (3)	0.0496 (8)	
H13C	0.8195	1.0495	0.7507	0.060*	
C14A	0.61745 (8)	0.85411 (17)	0.0434 (3)	0.0353 (6)	
H14A	0.6343	0.8996	0.0402	0.042*	
C14C	0.87728 (8)	0.68750 (18)	−0.0340 (2)	0.0346 (6)	
H14B	0.8607	0.7338	−0.0431	0.042*	
C14B	0.88061 (8)	1.00581 (17)	0.9191 (3)	0.0362 (6)	
H14C	0.8678	1.0563	0.9176	0.043*	
C15C	0.90579 (9)	0.6871 (2)	−0.1302 (3)	0.0464 (7)	
H15A	0.8987	0.6473	−0.1922	0.056*	
H15B	0.9057	0.7365	−0.1722	0.056*	
C15B	0.90755 (9)	1.0015 (2)	0.8189 (3)	0.0495 (8)	
H15C	0.8971	0.9659	0.7535	0.059*	
H15D	0.9104	1.0518	0.7819	0.059*	
C15A	0.59243 (9)	0.86225 (18)	0.1472 (3)	0.0411 (7)	
H15E	0.5843	0.8123	0.1751	0.049*	
H15F	0.6067	0.8895	0.2179	0.049*	
C16C	0.94574 (10)	0.6712 (3)	−0.0572 (3)	0.0643 (11)	
H16A	0.9507	0.6163	−0.0487	0.077*	
H16B	0.9668	0.6950	−0.0960	0.077*	
C16A	0.55793 (11)	0.9070 (3)	0.0899 (4)	0.0615 (10)	
H16C	0.5639	0.9617	0.0899	0.074*	
H16D	0.5351	0.8986	0.1333	0.074*	
C16B	0.94672 (10)	0.9736 (3)	0.8845 (4)	0.0686 (12)	
H16E	0.9478	0.9178	0.8882	0.082*	
H16F	0.9687	0.9927	0.8446	0.082*	
C17A	0.55103 (9)	0.8763 (3)	−0.0387 (3)	0.0564 (9)	
H17A	0.5322	0.8341	−0.0437	0.068*	
H17B	0.5405	0.9162	−0.0967	0.068*	
C17C	0.94190 (9)	0.7067 (3)	0.0640 (3)	0.0570 (10)	
H17C	0.9611	0.6854	0.1295	0.068*	
H17D	0.9456	0.7620	0.0608	0.068*	
C17B	0.94646 (9)	1.0081 (3)	1.0113 (4)	0.0625 (11)	
H17E	0.9654	0.9826	1.0729	0.075*	
H17F	0.9525	1.0627	1.0108	0.075*	
C18B	0.89627 (9)	1.04214 (17)	1.1372 (3)	0.0384 (6)	
H18A	0.8685	1.0350	1.1452	0.046*	
H18B	0.9004	1.0960	1.1187	0.046*	
C18C	0.88879 (9)	0.73210 (18)	0.1836 (3)	0.0394 (6)	
H18C	0.8600	0.7333	0.1718	0.047*	
H18D	0.8983	0.7846	0.1809	0.047*	
C18A	0.60099 (9)	0.88466 (19)	−0.1784 (3)	0.0429 (7)	
H18E	0.6284	0.8716	−0.1839	0.051*	
H18F	0.5991	0.9402	−0.1722	0.051*	
C19B	0.92066 (10)	1.02140 (18)	1.2559 (3)	0.0430 (7)	
H19A	0.9229	0.9658	1.2701	0.052*	
C19C	0.90315 (9)	0.69780 (19)	0.3067 (3)	0.0414 (7)	
H19B	0.8992	0.6420	0.3097	0.050*	
C19A	0.57583 (10)	0.85798 (19)	−0.2918 (3)	0.0450 (7)	
H19C	0.5707	0.8025	−0.2913	0.054*	
C20A	0.54190 (10)	0.9032 (2)	−0.3598 (3)	0.0479 (8)	
H20A	0.5192	0.8715	−0.3944	0.057*	
C20B	0.95673 (10)	1.0655 (2)	1.3112 (3)	0.0492 (8)	
H20B	0.9778	1.0330	1.3547	0.059*	
C20C	0.93935 (9)	0.72480 (19)	0.3894 (3)	0.0432 (7)	
H20C	0.9545	0.6838	0.4362	0.052*	
C21C	0.89832 (10)	0.7365 (2)	0.4234 (3)	0.0530 (9)	
C21A	0.57894 (12)	0.8878 (3)	−0.4164 (3)	0.0597 (10)	
C21B	0.91957 (12)	1.0658 (2)	1.3705 (3)	0.0541 (9)	
C22B	0.89454 (13)	1.1358 (3)	1.3665 (4)	0.0672 (11)	
H22A	0.8720	1.1260	1.4089	0.101*	
H22B	0.8857	1.1493	1.2810	0.101*	
H22C	0.9097	1.1775	1.4069	0.101*	
C22C	0.88819 (16)	0.6850 (4)	0.5251 (4)	0.0888 (17)	
H22D	0.8617	0.6958	0.5416	0.133*	
H22E	0.9065	0.6938	0.5995	0.133*	
H22F	0.8899	0.6323	0.4996	0.133*	
C22A	0.60725 (14)	0.9508 (3)	−0.4305 (5)	0.0899 (18)	
H22G	0.6291	0.9312	−0.4688	0.135*	
H22H	0.6169	0.9716	−0.3496	0.135*	
H22I	0.5941	0.9906	−0.4821	0.135*	
C23C	0.87987 (12)	0.8133 (3)	0.4212 (4)	0.0679 (11)	
H23A	0.8538	0.8091	0.4457	0.102*	
H23B	0.8779	0.8342	0.3382	0.102*	
H23C	0.8958	0.8467	0.4783	0.102*	
C23B	0.92173 (17)	1.0215 (3)	1.4887 (4)	0.0816 (14)	
H23D	0.8968	1.0248	1.5208	0.122*	
H23E	0.9423	1.0424	1.5489	0.122*	
H23F	0.9275	0.9685	1.4730	0.122*	
C23A	0.57365 (16)	0.8325 (3)	−0.5219 (4)	0.0868 (17)	
H23G	0.5984	0.8256	−0.5539	0.130*	
H23H	0.5542	0.8521	−0.5870	0.130*	
H23I	0.5648	0.7839	−0.4932	0.130*	
C24C	0.96302 (9)	0.78642 (19)	0.3501 (3)	0.0430 (7)	
C24A	0.53123 (11)	0.9789 (2)	−0.3215 (3)	0.0505 (8)	
C24B	0.97119 (10)	1.1316 (2)	1.2508 (3)	0.0510 (8)	
C25C	1.02614 (12)	0.8199 (3)	0.3171 (5)	0.0731 (12)	
H25A	1.0525	0.7993	0.3246	0.110*	
H25B	1.0260	0.8650	0.3686	0.110*	
H25C	1.0176	0.8334	0.2314	0.110*	
C25B	1.02795 (15)	1.1948 (3)	1.2119 (7)	0.107 (2)	
H25D	1.0563	1.1912	1.2305	0.161*	
H25E	1.0192	1.2426	1.2437	0.161*	
H25F	1.0205	1.1929	1.1230	0.161*	
C25A	0.47831 (16)	1.0591 (3)	−0.3037 (6)	0.0923 (17)	
H25G	0.4499	1.0596	−0.3208	0.139*	
H25H	0.4890	1.0997	−0.3497	0.139*	
H25I	0.4863	1.0666	−0.2158	0.139*	

### Atomic displacement parameters (Å^2^)

**Table:**

	*U*^11^	*U*^22^	*U*^33^	*U*^12^	*U*^13^	*U*^23^
O1A	0.0363 (11)	0.0367 (11)	0.0476 (12)	−0.0111 (8)	0.0026 (9)	−0.0030 (9)
O1C	0.0513 (13)	0.0406 (11)	0.0414 (11)	0.0158 (10)	0.0051 (9)	0.0032 (9)
O1B	0.0438 (12)	0.0351 (11)	0.0637 (15)	0.0087 (9)	0.0080 (10)	−0.0022 (10)
O2B	0.0548 (14)	0.0447 (13)	0.0797 (19)	−0.0020 (12)	−0.0036 (13)	0.0076 (13)
O2A	0.0625 (18)	0.0436 (15)	0.122 (3)	−0.0002 (13)	−0.0030 (17)	−0.0005 (16)
O2C	0.0548 (16)	0.0404 (14)	0.132 (3)	0.0018 (12)	0.0165 (16)	0.0139 (16)
O3A	0.0514 (14)	0.0556 (15)	0.0687 (17)	0.0116 (11)	0.0085 (12)	−0.0045 (13)
O3C	0.0402 (12)	0.0709 (17)	0.0567 (15)	0.0051 (11)	0.0075 (10)	0.0093 (13)
O3B	0.0432 (15)	0.0626 (17)	0.125 (3)	−0.0071 (13)	−0.0049 (16)	0.0134 (18)
N1C	0.0316 (11)	0.0566 (15)	0.0309 (12)	0.0061 (11)	0.0006 (9)	−0.0027 (11)
N1A	0.0275 (11)	0.0484 (14)	0.0379 (13)	−0.0046 (10)	0.0013 (9)	−0.0015 (11)
N1B	0.0298 (12)	0.0468 (14)	0.0410 (13)	0.0061 (10)	−0.0005 (10)	−0.0016 (11)
C1C	0.0343 (14)	0.0358 (14)	0.0321 (14)	0.0059 (11)	0.0033 (11)	0.0034 (11)
C1A	0.0281 (13)	0.0320 (13)	0.0414 (15)	−0.0067 (10)	0.0033 (11)	−0.0023 (11)
C1B	0.0331 (14)	0.0290 (13)	0.0467 (16)	0.0035 (10)	0.0040 (11)	−0.0024 (12)
C2A	0.0292 (13)	0.0383 (14)	0.0395 (15)	0.0002 (11)	0.0006 (11)	0.0005 (12)
C2B	0.0297 (13)	0.0352 (14)	0.0417 (15)	0.0000 (11)	0.0004 (11)	−0.0018 (12)
C2C	0.0373 (15)	0.0407 (15)	0.0330 (14)	−0.0035 (12)	0.0051 (11)	−0.0033 (12)
C3B	0.0347 (15)	0.0426 (16)	0.0533 (18)	0.0040 (12)	0.0043 (13)	0.0051 (14)
C3C	0.058 (2)	0.0418 (16)	0.0381 (16)	−0.0043 (14)	0.0097 (14)	0.0008 (13)
C3A	0.0310 (14)	0.0436 (16)	0.0539 (19)	−0.0046 (12)	0.0047 (13)	−0.0020 (14)
C4B	0.0375 (16)	0.057 (2)	0.0553 (19)	0.0105 (14)	0.0051 (14)	−0.0049 (16)
C4A	0.0305 (15)	0.0546 (19)	0.059 (2)	−0.0046 (13)	0.0066 (13)	0.0074 (16)
C4C	0.075 (3)	0.064 (2)	0.0426 (19)	−0.017 (2)	0.0197 (17)	−0.0008 (17)
C5A	0.0364 (16)	0.068 (2)	0.051 (2)	0.0043 (15)	0.0102 (14)	0.0079 (17)
C5B	0.0411 (17)	0.067 (2)	0.0468 (19)	−0.0021 (15)	0.0118 (14)	−0.0066 (16)
C5C	0.052 (2)	0.077 (3)	0.050 (2)	−0.0188 (19)	0.0189 (16)	−0.0196 (19)
C6C	0.0376 (16)	0.061 (2)	0.055 (2)	0.0003 (15)	0.0091 (14)	−0.0138 (17)
C6A	0.0518 (19)	0.055 (2)	0.0499 (19)	0.0058 (16)	0.0118 (15)	−0.0045 (16)
C6B	0.060 (2)	0.058 (2)	0.0458 (19)	−0.0088 (17)	0.0110 (16)	0.0048 (16)
C7B	0.0455 (17)	0.0380 (15)	0.0462 (18)	0.0003 (13)	0.0034 (13)	0.0025 (13)
C7C	0.0395 (15)	0.0445 (16)	0.0419 (16)	−0.0016 (13)	0.0051 (12)	−0.0014 (13)
C7A	0.0392 (15)	0.0414 (16)	0.0408 (16)	−0.0007 (12)	0.0039 (12)	−0.0005 (13)
C8B	0.0308 (14)	0.0472 (16)	0.0451 (17)	−0.0025 (12)	0.0059 (12)	−0.0080 (13)
C8A	0.0326 (14)	0.0393 (15)	0.0411 (16)	0.0015 (11)	0.0024 (11)	−0.0001 (12)
C8C	0.0362 (14)	0.0388 (15)	0.0351 (14)	−0.0019 (11)	0.0060 (11)	0.0003 (11)
C9C	0.0467 (18)	0.054 (2)	0.0430 (18)	0.0083 (15)	−0.0029 (14)	−0.0052 (15)
C9A	0.0483 (19)	0.058 (2)	0.049 (2)	−0.0133 (16)	−0.0106 (15)	0.0079 (16)
C9B	0.0508 (19)	0.056 (2)	0.053 (2)	−0.0104 (15)	0.0166 (15)	−0.0129 (16)
C10A	0.058 (2)	0.073 (3)	0.057 (2)	−0.0085 (19)	−0.0152 (18)	0.0033 (19)
C10B	0.066 (2)	0.083 (3)	0.053 (2)	−0.027 (2)	0.0202 (18)	−0.034 (2)
C10C	0.0454 (19)	0.073 (2)	0.0462 (19)	0.0050 (17)	−0.0057 (14)	−0.0041 (18)
C11C	0.0471 (19)	0.080 (3)	0.0355 (16)	−0.0170 (17)	0.0056 (13)	−0.0085 (16)
C11A	0.062 (2)	0.079 (3)	0.043 (2)	0.015 (2)	−0.0032 (16)	0.0041 (18)
C11B	0.054 (2)	0.106 (4)	0.043 (2)	−0.022 (2)	0.0061 (16)	−0.018 (2)
C12B	0.049 (2)	0.084 (3)	0.047 (2)	−0.0039 (19)	−0.0053 (16)	−0.0007 (19)
C12C	0.065 (2)	0.056 (2)	0.0466 (19)	−0.0077 (17)	0.0144 (16)	−0.0172 (16)
C12A	0.071 (2)	0.056 (2)	0.050 (2)	0.0044 (18)	0.0096 (17)	0.0093 (17)
C13C	0.058 (2)	0.0436 (17)	0.0406 (16)	0.0018 (14)	0.0106 (14)	−0.0033 (14)
C13A	0.0536 (19)	0.0441 (17)	0.0467 (18)	−0.0027 (14)	0.0092 (14)	0.0028 (14)
C13B	0.0459 (18)	0.054 (2)	0.0450 (18)	0.0022 (15)	−0.0065 (14)	−0.0070 (15)
C14A	0.0288 (13)	0.0388 (15)	0.0375 (15)	−0.0050 (11)	0.0013 (11)	−0.0013 (11)
C14C	0.0316 (13)	0.0428 (15)	0.0286 (13)	0.0001 (12)	0.0012 (10)	−0.0004 (11)
C14B	0.0311 (14)	0.0414 (15)	0.0349 (14)	−0.0018 (11)	−0.0002 (10)	0.0016 (11)
C15C	0.0425 (16)	0.064 (2)	0.0334 (15)	−0.0085 (15)	0.0067 (12)	−0.0019 (15)
C15B	0.0390 (16)	0.067 (2)	0.0436 (17)	−0.0078 (15)	0.0086 (13)	−0.0021 (15)
C15A	0.0387 (15)	0.0450 (17)	0.0405 (16)	−0.0044 (12)	0.0087 (12)	−0.0039 (13)
C16C	0.0371 (17)	0.111 (4)	0.046 (2)	−0.0007 (19)	0.0119 (14)	−0.013 (2)
C16A	0.047 (2)	0.082 (3)	0.057 (2)	0.0123 (18)	0.0121 (16)	0.001 (2)
C16B	0.0345 (17)	0.107 (4)	0.065 (2)	−0.0014 (19)	0.0121 (16)	−0.020 (2)
C17A	0.0293 (16)	0.086 (3)	0.052 (2)	0.0042 (16)	0.0011 (13)	−0.0020 (19)
C17C	0.0313 (15)	0.097 (3)	0.0416 (17)	−0.0010 (16)	0.0021 (12)	−0.0098 (18)
C17B	0.0284 (16)	0.097 (3)	0.061 (2)	−0.0005 (17)	−0.0006 (14)	−0.012 (2)
C18B	0.0392 (15)	0.0353 (14)	0.0389 (15)	0.0026 (11)	−0.0013 (12)	−0.0015 (11)
C18C	0.0382 (15)	0.0487 (17)	0.0306 (14)	0.0031 (12)	0.0021 (11)	−0.0033 (12)
C18A	0.0344 (15)	0.0462 (17)	0.0474 (18)	0.0016 (12)	0.0027 (12)	0.0074 (14)
C19B	0.0513 (18)	0.0361 (15)	0.0388 (15)	−0.0029 (13)	−0.0041 (13)	0.0017 (12)
C19C	0.0430 (16)	0.0449 (17)	0.0360 (15)	−0.0063 (13)	0.0035 (12)	0.0027 (13)
C19A	0.0471 (17)	0.0455 (17)	0.0414 (17)	0.0100 (14)	0.0029 (13)	0.0039 (13)
C20A	0.0475 (18)	0.0496 (18)	0.0447 (18)	0.0056 (14)	−0.0002 (14)	0.0057 (14)
C20B	0.0498 (19)	0.0471 (18)	0.0472 (18)	−0.0001 (14)	−0.0070 (14)	0.0005 (14)
C20C	0.0468 (17)	0.0500 (18)	0.0302 (14)	−0.0024 (14)	−0.0049 (12)	0.0039 (13)
C21C	0.0491 (19)	0.075 (2)	0.0353 (17)	−0.0121 (17)	0.0066 (13)	−0.0034 (16)
C21A	0.065 (2)	0.071 (2)	0.0453 (19)	0.024 (2)	0.0139 (16)	0.0149 (18)
C21B	0.070 (2)	0.051 (2)	0.0382 (17)	−0.0082 (17)	−0.0016 (15)	−0.0033 (15)
C22B	0.071 (3)	0.066 (3)	0.066 (2)	−0.002 (2)	0.012 (2)	−0.021 (2)
C22C	0.104 (4)	0.126 (4)	0.039 (2)	−0.045 (3)	0.019 (2)	−0.001 (2)
C22A	0.070 (3)	0.112 (4)	0.092 (4)	0.022 (3)	0.030 (3)	0.055 (3)
C23C	0.052 (2)	0.089 (3)	0.065 (3)	0.002 (2)	0.0129 (18)	−0.028 (2)
C23B	0.115 (4)	0.086 (3)	0.041 (2)	−0.022 (3)	0.000 (2)	0.000 (2)
C23A	0.114 (4)	0.107 (4)	0.039 (2)	0.054 (3)	0.010 (2)	0.006 (2)
C24C	0.0399 (16)	0.0459 (17)	0.0418 (17)	−0.0015 (13)	0.0000 (12)	−0.0039 (14)
C24A	0.054 (2)	0.0438 (18)	0.053 (2)	0.0047 (15)	0.0059 (15)	0.0061 (15)
C24B	0.0481 (19)	0.0459 (18)	0.056 (2)	−0.0003 (14)	−0.0051 (15)	−0.0046 (15)
C25C	0.046 (2)	0.098 (3)	0.077 (3)	−0.011 (2)	0.0140 (19)	0.010 (3)
C25B	0.059 (3)	0.076 (3)	0.185 (7)	−0.022 (3)	0.015 (3)	0.026 (4)
C25A	0.086 (4)	0.071 (3)	0.127 (5)	0.024 (3)	0.039 (3)	−0.013 (3)

### Geometric parameters (Å, °)

**Table:**

O1A—C1A	1.416 (3)	C12A—H12C	0.9500
O1A—H1A	0.8400	C13C—H13A	0.9500
O1C—C1C	1.417 (3)	C13A—H13B	0.9500
O1C—H1C	0.8400	C13B—H13C	0.9500
O1B—C1B	1.425 (3)	C14A—C15A	1.532 (4)
O1B—H1BA	0.8552	C14A—H14A	1.0000
O2B—C24B	1.202 (4)	C14C—C15C	1.543 (4)
O2A—C24A	1.210 (5)	C14C—H14B	1.0000
O2C—C24C	1.185 (4)	C14B—C15B	1.540 (4)
O3A—C24A	1.333 (4)	C14B—H14C	1.0000
O3A—C25A	1.443 (5)	C15C—C16C	1.535 (5)
O3C—C24C	1.344 (4)	C15C—H15A	0.9900
O3C—C25C	1.438 (5)	C15C—H15B	0.9900
O3B—C24B	1.345 (4)	C15B—C16B	1.536 (5)
O3B—C25B	1.440 (6)	C15B—H15C	0.9900
N1C—C17C	1.455 (4)	C15B—H15D	0.9900
N1C—C18C	1.466 (4)	C15A—C16A	1.505 (5)
N1C—C14C	1.475 (3)	C15A—H15E	0.9900
N1A—C18A	1.465 (4)	C15A—H15F	0.9900
N1A—C14A	1.478 (4)	C16C—C17C	1.497 (5)
N1A—C17A	1.478 (4)	C16C—H16A	0.9900
N1B—C17B	1.466 (4)	C16C—H16B	0.9900
N1B—C18B	1.469 (4)	C16A—C17A	1.506 (6)
N1B—C14B	1.486 (4)	C16A—H16C	0.9900
C1C—C8C	1.524 (4)	C16A—H16D	0.9900
C1C—C2C	1.550 (4)	C16B—C17B	1.525 (6)
C1C—C14C	1.567 (4)	C16B—H16E	0.9900
C1A—C8A	1.522 (4)	C16B—H16F	0.9900
C1A—C2A	1.536 (4)	C17A—H17A	0.9900
C1A—C14A	1.564 (4)	C17A—H17B	0.9900
C1B—C8B	1.533 (4)	C17C—H17C	0.9900
C1B—C2B	1.543 (4)	C17C—H17D	0.9900
C1B—C14B	1.554 (4)	C17B—H17E	0.9900
C2A—C3A	1.395 (4)	C17B—H17F	0.9900
C2A—C7A	1.395 (4)	C18B—C19B	1.507 (4)
C2B—C7B	1.382 (4)	C18B—H18A	0.9900
C2B—C3B	1.386 (4)	C18B—H18B	0.9900
C2C—C7C	1.392 (5)	C18C—C19C	1.509 (4)
C2C—C3C	1.394 (4)	C18C—H18C	0.9900
C3B—C4B	1.390 (5)	C18C—H18D	0.9900
C3B—H3BA	0.9500	C18A—C19A	1.503 (5)
C3C—C4C	1.386 (5)	C18A—H18E	0.9900
C3C—H3CA	0.9500	C18A—H18F	0.9900
C3A—C4A	1.398 (5)	C19B—C21B	1.492 (5)
C3A—H3AA	0.9500	C19B—C20B	1.533 (4)
C4B—C5B	1.376 (5)	C19B—H19A	1.0000
C4B—H4BA	0.9500	C19C—C21C	1.486 (5)
C4A—C5A	1.384 (5)	C19C—C20C	1.528 (4)
C4A—H4AA	0.9500	C19C—H19B	1.0000
C4C—C5C	1.385 (6)	C19A—C21A	1.487 (5)
C4C—H4CA	0.9500	C19A—C20A	1.536 (4)
C5A—C6A	1.380 (5)	C19A—H19C	1.0000
C5A—H5AA	0.9500	C20A—C24A	1.470 (5)
C5B—C6B	1.375 (5)	C20A—C21A	1.527 (5)
C5B—H5BA	0.9500	C20A—H20A	1.0000
C5C—C6C	1.386 (6)	C20B—C24B	1.468 (5)
C5C—H5CA	0.9500	C20B—C21B	1.522 (6)
C6C—C7C	1.388 (4)	C20B—H20B	1.0000
C6C—H6CA	0.9500	C20C—C24C	1.467 (5)
C6A—C7A	1.390 (5)	C20C—C21C	1.535 (5)
C6A—H6AA	0.9500	C20C—H20C	1.0000
C6B—C7B	1.396 (5)	C21C—C23C	1.504 (6)
C6B—H6BA	0.9500	C21C—C22C	1.522 (6)
C7B—H7BA	0.9500	C21A—C22A	1.511 (7)
C7C—H7CA	0.9500	C21A—C23A	1.512 (6)
C7A—H7AA	0.9500	C21B—C22B	1.513 (6)
C8B—C9B	1.381 (5)	C21B—C23B	1.513 (6)
C8B—C13B	1.387 (5)	C22B—H22A	0.9800
C8A—C13A	1.384 (4)	C22B—H22B	0.9800
C8A—C9A	1.395 (4)	C22B—H22C	0.9800
C8C—C13C	1.382 (4)	C22C—H22D	0.9800
C8C—C9C	1.390 (4)	C22C—H22E	0.9800
C9C—C10C	1.383 (5)	C22C—H22F	0.9800
C9C—H9CA	0.9500	C22A—H22G	0.9800
C9A—C10A	1.388 (5)	C22A—H22H	0.9800
C9A—H9AA	0.9500	C22A—H22I	0.9800
C9B—C10B	1.399 (6)	C23C—H23A	0.9800
C9B—H9BA	0.9500	C23C—H23B	0.9800
C10A—C11A	1.382 (6)	C23C—H23C	0.9800
C10A—H10A	0.9500	C23B—H23D	0.9800
C10B—C11B	1.365 (7)	C23B—H23E	0.9800
C10B—H10B	0.9500	C23B—H23F	0.9800
C10C—C11C	1.369 (6)	C23A—H23G	0.9800
C10C—H10C	0.9500	C23A—H23H	0.9800
C11C—C12C	1.382 (6)	C23A—H23I	0.9800
C11C—H11A	0.9500	C25C—H25A	0.9800
C11A—C12A	1.371 (6)	C25C—H25B	0.9800
C11A—H11B	0.9500	C25C—H25C	0.9800
C11B—C12B	1.371 (6)	C25B—H25D	0.9800
C11B—H11C	0.9500	C25B—H25E	0.9800
C12B—C13B	1.390 (5)	C25B—H25F	0.9800
C12B—H12A	0.9500	C25A—H25G	0.9800
C12C—C13C	1.388 (5)	C25A—H25H	0.9800
C12C—H12B	0.9500	C25A—H25I	0.9800
C12A—C13A	1.392 (5)		
			
C1A—O1A—H1A	109.5	C16A—C15A—H15F	111.0
C1C—O1C—H1C	109.5	C14A—C15A—H15F	111.0
C1B—O1B—H1BA	97.3	H15E—C15A—H15F	109.0
C24A—O3A—C25A	115.7 (4)	C17C—C16C—C15C	102.0 (3)
C24C—O3C—C25C	114.9 (3)	C17C—C16C—H16A	111.4
C24B—O3B—C25B	115.5 (4)	C15C—C16C—H16A	111.4
C17C—N1C—C18C	113.7 (3)	C17C—C16C—H16B	111.4
C17C—N1C—C14C	108.8 (2)	C15C—C16C—H16B	111.4
C18C—N1C—C14C	116.8 (2)	H16A—C16C—H16B	109.2
C18A—N1A—C14A	115.8 (2)	C15A—C16A—C17A	102.9 (3)
C18A—N1A—C17A	114.2 (3)	C15A—C16A—H16C	111.2
C14A—N1A—C17A	108.3 (2)	C17A—C16A—H16C	111.2
C17B—N1B—C18B	112.7 (3)	C15A—C16A—H16D	111.2
C17B—N1B—C14B	107.3 (3)	C17A—C16A—H16D	111.2
C18B—N1B—C14B	113.9 (2)	H16C—C16A—H16D	109.1
O1C—C1C—C8C	107.5 (2)	C17B—C16B—C15B	100.9 (3)
O1C—C1C—C2C	110.0 (2)	C17B—C16B—H16E	111.6
C8C—C1C—C2C	110.6 (2)	C15B—C16B—H16E	111.6
O1C—C1C—C14C	106.7 (2)	C17B—C16B—H16F	111.6
C8C—C1C—C14C	111.3 (2)	C15B—C16B—H16F	111.6
C2C—C1C—C14C	110.6 (2)	H16E—C16B—H16F	109.4
O1A—C1A—C8A	107.4 (2)	N1A—C17A—C16A	106.8 (3)
O1A—C1A—C2A	110.2 (2)	N1A—C17A—H17A	110.4
C8A—C1A—C2A	111.0 (2)	C16A—C17A—H17A	110.4
O1A—C1A—C14A	106.8 (2)	N1A—C17A—H17B	110.4
C8A—C1A—C14A	110.3 (2)	C16A—C17A—H17B	110.4
C2A—C1A—C14A	111.1 (2)	H17A—C17A—H17B	108.6
O1B—C1B—C8B	107.4 (2)	N1C—C17C—C16C	104.1 (3)
O1B—C1B—C2B	110.0 (2)	N1C—C17C—H17C	110.9
C8B—C1B—C2B	110.0 (2)	C16C—C17C—H17C	110.9
O1B—C1B—C14B	106.4 (2)	N1C—C17C—H17D	110.9
C8B—C1B—C14B	111.2 (3)	C16C—C17C—H17D	110.9
C2B—C1B—C14B	111.8 (2)	H17C—C17C—H17D	109.0
C3A—C2A—C7A	117.8 (3)	N1B—C17B—C16B	103.3 (3)
C3A—C2A—C1A	122.4 (3)	N1B—C17B—H17E	111.1
C7A—C2A—C1A	119.8 (3)	C16B—C17B—H17E	111.1
C7B—C2B—C3B	118.2 (3)	N1B—C17B—H17F	111.1
C7B—C2B—C1B	120.6 (3)	C16B—C17B—H17F	111.1
C3B—C2B—C1B	121.2 (3)	H17E—C17B—H17F	109.1
C7C—C2C—C3C	117.8 (3)	N1B—C18B—C19B	110.6 (2)
C7C—C2C—C1C	122.4 (3)	N1B—C18B—H18A	109.5
C3C—C2C—C1C	119.8 (3)	C19B—C18B—H18A	109.5
C2B—C3B—C4B	121.2 (3)	N1B—C18B—H18B	109.5
C2B—C3B—H3BA	119.4	C19B—C18B—H18B	109.5
C4B—C3B—H3BA	119.4	H18A—C18B—H18B	108.1
C4C—C3C—C2C	120.8 (3)	N1C—C18C—C19C	110.6 (3)
C4C—C3C—H3CA	119.6	N1C—C18C—H18C	109.5
C2C—C3C—H3CA	119.6	C19C—C18C—H18C	109.5
C2A—C3A—C4A	121.0 (3)	N1C—C18C—H18D	109.5
C2A—C3A—H3AA	119.5	C19C—C18C—H18D	109.5
C4A—C3A—H3AA	119.5	H18C—C18C—H18D	108.1
C5B—C4B—C3B	120.1 (3)	N1A—C18A—C19A	110.8 (3)
C5B—C4B—H4BA	119.9	N1A—C18A—H18E	109.5
C3B—C4B—H4BA	119.9	C19A—C18A—H18E	109.5
C5A—C4A—C3A	120.0 (3)	N1A—C18A—H18F	109.5
C5A—C4A—H4AA	120.0	C19A—C18A—H18F	109.5
C3A—C4A—H4AA	120.0	H18E—C18A—H18F	108.1
C5C—C4C—C3C	121.0 (4)	C21B—C19B—C18B	122.1 (3)
C5C—C4C—H4CA	119.5	C21B—C19B—C20B	60.4 (2)
C3C—C4C—H4CA	119.5	C18B—C19B—C20B	123.8 (3)
C6A—C5A—C4A	119.6 (3)	C21B—C19B—H19A	113.5
C6A—C5A—H5AA	120.2	C18B—C19B—H19A	113.5
C4A—C5A—H5AA	120.2	C20B—C19B—H19A	113.5
C6B—C5B—C4B	119.4 (3)	C21C—C19C—C18C	121.9 (3)
C6B—C5B—H5BA	120.3	C21C—C19C—C20C	61.2 (2)
C4B—C5B—H5BA	120.3	C18C—C19C—C20C	123.8 (3)
C4C—C5C—C6C	118.8 (3)	C21C—C19C—H19B	113.4
C4C—C5C—H5CA	120.6	C18C—C19C—H19B	113.4
C6C—C5C—H5CA	120.6	C20C—C19C—H19B	113.4
C5C—C6C—C7C	120.3 (3)	C21A—C19A—C18A	123.4 (3)
C5C—C6C—H6CA	119.9	C21A—C19A—C20A	60.6 (2)
C7C—C6C—H6CA	119.9	C18A—C19A—C20A	124.4 (3)
C5A—C6A—C7A	120.3 (3)	C21A—C19A—H19C	112.9
C5A—C6A—H6AA	119.9	C18A—C19A—H19C	112.9
C7A—C6A—H6AA	119.9	C20A—C19A—H19C	112.9
C5B—C6B—C7B	120.4 (3)	C24A—C20A—C21A	122.6 (3)
C5B—C6B—H6BA	119.8	C24A—C20A—C19A	122.9 (3)
C7B—C6B—H6BA	119.8	C21A—C20A—C19A	58.1 (2)
C2B—C7B—C6B	120.7 (3)	C24A—C20A—H20A	114.0
C2B—C7B—H7BA	119.6	C21A—C20A—H20A	114.0
C6B—C7B—H7BA	119.6	C19A—C20A—H20A	114.0
C6C—C7C—C2C	121.4 (3)	C24B—C20B—C21B	123.5 (3)
C6C—C7C—H7CA	119.3	C24B—C20B—C19B	122.5 (3)
C2C—C7C—H7CA	119.3	C21B—C20B—C19B	58.5 (2)
C6A—C7A—C2A	121.3 (3)	C24B—C20B—H20B	113.8
C6A—C7A—H7AA	119.4	C21B—C20B—H20B	113.8
C2A—C7A—H7AA	119.4	C19B—C20B—H20B	113.8
C9B—C8B—C13B	118.6 (3)	C24C—C20C—C19C	120.5 (3)
C9B—C8B—C1B	120.5 (3)	C24C—C20C—C21C	123.1 (3)
C13B—C8B—C1B	121.0 (3)	C19C—C20C—C21C	58.0 (2)
C13A—C8A—C9A	117.9 (3)	C24C—C20C—H20C	114.6
C13A—C8A—C1A	120.5 (3)	C19C—C20C—H20C	114.6
C9A—C8A—C1A	121.6 (3)	C21C—C20C—H20C	114.6
C13C—C8C—C9C	117.5 (3)	C19C—C21C—C23C	120.1 (3)
C13C—C8C—C1C	120.7 (3)	C19C—C21C—C22C	115.0 (4)
C9C—C8C—C1C	121.7 (3)	C23C—C21C—C22C	114.6 (4)
C10C—C9C—C8C	120.7 (3)	C19C—C21C—C20C	60.7 (2)
C10C—C9C—H9CA	119.7	C23C—C21C—C20C	121.7 (3)
C8C—C9C—H9CA	119.7	C22C—C21C—C20C	114.3 (4)
C10A—C9A—C8A	120.9 (4)	C19A—C21A—C22A	119.0 (4)
C10A—C9A—H9AA	119.5	C19A—C21A—C23A	117.5 (4)
C8A—C9A—H9AA	119.5	C22A—C21A—C23A	114.5 (4)
C8B—C9B—C10B	119.7 (4)	C19A—C21A—C20A	61.2 (2)
C8B—C9B—H9BA	120.1	C22A—C21A—C20A	120.3 (4)
C10B—C9B—H9BA	120.1	C23A—C21A—C20A	114.0 (4)
C11A—C10A—C9A	120.3 (4)	C19B—C21B—C22B	119.1 (3)
C11A—C10A—H10A	119.9	C19B—C21B—C23B	116.7 (3)
C9A—C10A—H10A	119.9	C22B—C21B—C23B	114.5 (4)
C11B—C10B—C9B	121.2 (4)	C19B—C21B—C20B	61.1 (2)
C11B—C10B—H10B	119.4	C22B—C21B—C20B	120.5 (3)
C9B—C10B—H10B	119.4	C23B—C21B—C20B	114.8 (4)
C11C—C10C—C9C	121.3 (4)	C21B—C22B—H22A	109.5
C11C—C10C—H10C	119.3	C21B—C22B—H22B	109.5
C9C—C10C—H10C	119.3	H22A—C22B—H22B	109.5
C10C—C11C—C12C	118.8 (3)	C21B—C22B—H22C	109.5
C10C—C11C—H11A	120.6	H22A—C22B—H22C	109.5
C12C—C11C—H11A	120.6	H22B—C22B—H22C	109.5
C12A—C11A—C10A	119.4 (4)	C21C—C22C—H22D	109.5
C12A—C11A—H11B	120.3	C21C—C22C—H22E	109.5
C10A—C11A—H11B	120.3	H22D—C22C—H22E	109.5
C10B—C11B—C12B	119.4 (4)	C21C—C22C—H22F	109.5
C10B—C11B—H11C	120.3	H22D—C22C—H22F	109.5
C12B—C11B—H11C	120.3	H22E—C22C—H22F	109.5
C11B—C12B—C13B	120.1 (4)	C21A—C22A—H22G	109.5
C11B—C12B—H12A	120.0	C21A—C22A—H22H	109.5
C13B—C12B—H12A	120.0	H22G—C22A—H22H	109.5
C11C—C12C—C13C	119.9 (3)	C21A—C22A—H22I	109.5
C11C—C12C—H12B	120.0	H22G—C22A—H22I	109.5
C13C—C12C—H12B	120.0	H22H—C22A—H22I	109.5
C11A—C12A—C13A	120.5 (4)	C21C—C23C—H23A	109.5
C11A—C12A—H12C	119.7	C21C—C23C—H23B	109.5
C13A—C12A—H12C	119.7	H23A—C23C—H23B	109.5
C8C—C13C—C12C	121.7 (3)	C21C—C23C—H23C	109.5
C8C—C13C—H13A	119.1	H23A—C23C—H23C	109.5
C12C—C13C—H13A	119.1	H23B—C23C—H23C	109.5
C8A—C13A—C12A	121.0 (3)	C21B—C23B—H23D	109.5
C8A—C13A—H13B	119.5	C21B—C23B—H23E	109.5
C12A—C13A—H13B	119.5	H23D—C23B—H23E	109.5
C8B—C13B—C12B	121.0 (3)	C21B—C23B—H23F	109.5
C8B—C13B—H13C	119.5	H23D—C23B—H23F	109.5
C12B—C13B—H13C	119.5	H23E—C23B—H23F	109.5
N1A—C14A—C15A	104.2 (2)	C21A—C23A—H23G	109.5
N1A—C14A—C1A	109.1 (2)	C21A—C23A—H23H	109.5
C15A—C14A—C1A	113.3 (2)	H23G—C23A—H23H	109.5
N1A—C14A—H14A	110.0	C21A—C23A—H23I	109.5
C15A—C14A—H14A	110.0	H23G—C23A—H23I	109.5
C1A—C14A—H14A	110.0	H23H—C23A—H23I	109.5
N1C—C14C—C15C	104.5 (2)	O2C—C24C—O3C	121.0 (3)
N1C—C14C—C1C	107.8 (2)	O2C—C24C—C20C	129.0 (3)
C15C—C14C—C1C	113.4 (2)	O3C—C24C—C20C	110.0 (3)
N1C—C14C—H14B	110.3	O2A—C24A—O3A	121.9 (3)
C15C—C14C—H14B	110.3	O2A—C24A—C20A	127.9 (4)
C1C—C14C—H14B	110.3	O3A—C24A—C20A	110.1 (3)
N1B—C14B—C15B	105.1 (2)	O2B—C24B—O3B	121.6 (4)
N1B—C14B—C1B	108.2 (2)	O2B—C24B—C20B	128.1 (3)
C15B—C14B—C1B	113.0 (3)	O3B—C24B—C20B	110.3 (3)
N1B—C14B—H14C	110.2	O3C—C25C—H25A	109.5
C15B—C14B—H14C	110.2	O3C—C25C—H25B	109.5
C1B—C14B—H14C	110.2	H25A—C25C—H25B	109.5
C16C—C15C—C14C	105.0 (3)	O3C—C25C—H25C	109.5
C16C—C15C—H15A	110.7	H25A—C25C—H25C	109.5
C14C—C15C—H15A	110.7	H25B—C25C—H25C	109.5
C16C—C15C—H15B	110.7	O3B—C25B—H25D	109.5
C14C—C15C—H15B	110.7	O3B—C25B—H25E	109.5
H15A—C15C—H15B	108.8	H25D—C25B—H25E	109.5
C16B—C15B—C14B	105.3 (3)	O3B—C25B—H25F	109.5
C16B—C15B—H15C	110.7	H25D—C25B—H25F	109.5
C14B—C15B—H15C	110.7	H25E—C25B—H25F	109.5
C16B—C15B—H15D	110.7	O3A—C25A—H25G	109.5
C14B—C15B—H15D	110.7	O3A—C25A—H25H	109.5
H15C—C15B—H15D	108.8	H25G—C25A—H25H	109.5
C16A—C15A—C14A	103.6 (3)	O3A—C25A—H25I	109.5
C16A—C15A—H15E	111.0	H25G—C25A—H25I	109.5
C14A—C15A—H15E	111.0	H25H—C25A—H25I	109.5
			
O1A—C1A—C2A—C3A	164.3 (3)	O1C—C1C—C14C—C15C	66.5 (3)
C8A—C1A—C2A—C3A	−76.9 (3)	C8C—C1C—C14C—C15C	−50.6 (3)
C14A—C1A—C2A—C3A	46.2 (4)	C2C—C1C—C14C—C15C	−174.0 (2)
O1A—C1A—C2A—C7A	−14.8 (4)	C17B—N1B—C14B—C15B	16.6 (4)
C8A—C1A—C2A—C7A	104.0 (3)	C18B—N1B—C14B—C15B	142.0 (3)
C14A—C1A—C2A—C7A	−132.9 (3)	C17B—N1B—C14B—C1B	137.6 (3)
O1B—C1B—C2B—C7B	−2.5 (4)	C18B—N1B—C14B—C1B	−97.0 (3)
C8B—C1B—C2B—C7B	115.5 (3)	O1B—C1B—C14B—N1B	−49.2 (3)
C14B—C1B—C2B—C7B	−120.5 (3)	C8B—C1B—C14B—N1B	−165.8 (2)
O1B—C1B—C2B—C3B	176.5 (3)	C2B—C1B—C14B—N1B	70.9 (3)
C8B—C1B—C2B—C3B	−65.4 (4)	O1B—C1B—C14B—C15B	66.7 (3)
C14B—C1B—C2B—C3B	58.5 (4)	C8B—C1B—C14B—C15B	−49.9 (3)
O1C—C1C—C2C—C7C	168.5 (3)	C2B—C1B—C14B—C15B	−173.2 (3)
C8C—C1C—C2C—C7C	−72.9 (4)	N1C—C14C—C15C—C16C	11.4 (4)
C14C—C1C—C2C—C7C	50.9 (4)	C1C—C14C—C15C—C16C	−105.7 (3)
O1C—C1C—C2C—C3C	−11.1 (4)	N1B—C14B—C15B—C16B	10.5 (4)
C8C—C1C—C2C—C3C	107.5 (3)	C1B—C14B—C15B—C16B	−107.2 (3)
C14C—C1C—C2C—C3C	−128.7 (3)	N1A—C14A—C15A—C16A	−33.1 (3)
C7B—C2B—C3B—C4B	−0.1 (5)	C1A—C14A—C15A—C16A	−151.6 (3)
C1B—C2B—C3B—C4B	−179.2 (3)	C14C—C15C—C16C—C17C	−31.1 (4)
C7C—C2C—C3C—C4C	−1.0 (5)	C14A—C15A—C16A—C17A	37.4 (4)
C1C—C2C—C3C—C4C	178.6 (3)	C14B—C15B—C16B—C17B	−32.1 (4)
C7A—C2A—C3A—C4A	1.4 (5)	C18A—N1A—C17A—C16A	−123.1 (3)
C1A—C2A—C3A—C4A	−177.6 (3)	C14A—N1A—C17A—C16A	7.6 (4)
C2B—C3B—C4B—C5B	0.5 (5)	C15A—C16A—C17A—N1A	−28.2 (4)
C2A—C3A—C4A—C5A	0.3 (5)	C18C—N1C—C17C—C16C	−166.1 (3)
C2C—C3C—C4C—C5C	0.2 (6)	C14C—N1C—C17C—C16C	−34.1 (4)
C3A—C4A—C5A—C6A	−1.7 (5)	C15C—C16C—C17C—N1C	39.6 (4)
C3B—C4B—C5B—C6B	−0.5 (5)	C18B—N1B—C17B—C16B	−163.7 (3)
C3C—C4C—C5C—C6C	0.9 (6)	C14B—N1B—C17B—C16B	−37.5 (4)
C4C—C5C—C6C—C7C	−1.2 (5)	C15B—C16B—C17B—N1B	42.4 (4)
C4A—C5A—C6A—C7A	1.2 (5)	C17B—N1B—C18B—C19B	−64.5 (4)
C4B—C5B—C6B—C7B	0.2 (5)	C14B—N1B—C18B—C19B	173.0 (3)
C3B—C2B—C7B—C6B	−0.2 (5)	C17C—N1C—C18C—C19C	−82.3 (3)
C1B—C2B—C7B—C6B	178.9 (3)	C14C—N1C—C18C—C19C	149.7 (3)
C5B—C6B—C7B—C2B	0.1 (5)	C14A—N1A—C18A—C19A	163.5 (3)
C5C—C6C—C7C—C2C	0.4 (5)	C17A—N1A—C18A—C19A	−69.6 (4)
C3C—C2C—C7C—C6C	0.7 (5)	N1B—C18B—C19B—C21B	172.8 (3)
C1C—C2C—C7C—C6C	−178.9 (3)	N1B—C18B—C19B—C20B	99.2 (4)
C5A—C6A—C7A—C2A	0.7 (5)	N1C—C18C—C19C—C21C	171.3 (3)
C3A—C2A—C7A—C6A	−1.9 (5)	N1C—C18C—C19C—C20C	96.6 (4)
C1A—C2A—C7A—C6A	177.2 (3)	N1A—C18A—C19A—C21A	175.2 (3)
O1B—C1B—C8B—C9B	18.7 (4)	N1A—C18A—C19A—C20A	100.3 (4)
C2B—C1B—C8B—C9B	−101.0 (3)	C21A—C19A—C20A—C24A	−110.7 (4)
C14B—C1B—C8B—C9B	134.7 (3)	C18A—C19A—C20A—C24A	1.6 (5)
O1B—C1B—C8B—C13B	−160.5 (3)	C18A—C19A—C20A—C21A	112.3 (4)
C2B—C1B—C8B—C13B	79.8 (4)	C21B—C19B—C20B—C24B	−112.1 (4)
C14B—C1B—C8B—C13B	−44.5 (4)	C18B—C19B—C20B—C24B	−1.3 (5)
O1A—C1A—C8A—C13A	−1.8 (4)	C18B—C19B—C20B—C21B	110.8 (4)
C2A—C1A—C8A—C13A	−122.3 (3)	C21C—C19C—C20C—C24C	−112.3 (4)
C14A—C1A—C8A—C13A	114.1 (3)	C18C—C19C—C20C—C24C	−1.3 (5)
O1A—C1A—C8A—C9A	−179.0 (3)	C18C—C19C—C20C—C21C	111.0 (4)
C2A—C1A—C8A—C9A	60.5 (4)	C18C—C19C—C21C—C23C	−2.1 (5)
C14A—C1A—C8A—C9A	−63.1 (4)	C20C—C19C—C21C—C23C	111.9 (4)
O1C—C1C—C8C—C13C	0.5 (4)	C18C—C19C—C21C—C22C	141.1 (4)
C2C—C1C—C8C—C13C	−119.6 (3)	C20C—C19C—C21C—C22C	−105.0 (4)
C14C—C1C—C8C—C13C	117.0 (3)	C18C—C19C—C21C—C20C	−113.9 (3)
O1C—C1C—C8C—C9C	−177.5 (3)	C24C—C20C—C21C—C19C	107.8 (3)
C2C—C1C—C8C—C9C	62.4 (4)	C24C—C20C—C21C—C23C	−1.4 (5)
C14C—C1C—C8C—C9C	−61.0 (4)	C19C—C20C—C21C—C23C	−109.2 (4)
C13C—C8C—C9C—C10C	0.4 (5)	C24C—C20C—C21C—C22C	−146.0 (3)
C1C—C8C—C9C—C10C	178.5 (3)	C19C—C20C—C21C—C22C	106.2 (4)
C13A—C8A—C9A—C10A	−0.4 (6)	C18A—C19A—C21A—C22A	−3.3 (5)
C1A—C8A—C9A—C10A	176.8 (4)	C20A—C19A—C21A—C22A	110.7 (4)
C13B—C8B—C9B—C10B	0.2 (5)	C18A—C19A—C21A—C23A	142.3 (4)
C1B—C8B—C9B—C10B	−179.1 (3)	C20A—C19A—C21A—C23A	−103.7 (4)
C8A—C9A—C10A—C11A	−0.7 (7)	C18A—C19A—C21A—C20A	−114.0 (4)
C8B—C9B—C10B—C11B	0.1 (6)	C24A—C20A—C21A—C19A	111.2 (4)
C8C—C9C—C10C—C11C	−0.7 (6)	C24A—C20A—C21A—C22A	2.5 (5)
C9C—C10C—C11C—C12C	0.9 (6)	C19A—C20A—C21A—C22A	−108.7 (4)
C9A—C10A—C11A—C12A	1.3 (7)	C24A—C20A—C21A—C23A	−139.4 (4)
C9B—C10B—C11B—C12B	−0.2 (6)	C19A—C20A—C21A—C23A	109.5 (4)
C10B—C11B—C12B—C13B	0.1 (6)	C18B—C19B—C21B—C22B	−2.7 (5)
C10C—C11C—C12C—C13C	−0.9 (5)	C20B—C19B—C21B—C22B	110.8 (4)
C10A—C11A—C12A—C13A	−0.8 (6)	C18B—C19B—C21B—C23B	141.5 (4)
C9C—C8C—C13C—C12C	−0.4 (5)	C20B—C19B—C21B—C23B	−105.0 (4)
C1C—C8C—C13C—C12C	−178.5 (3)	C18B—C19B—C21B—C20B	−113.5 (3)
C11C—C12C—C13C—C8C	0.7 (5)	C24B—C20B—C21B—C19B	110.5 (4)
C9A—C8A—C13A—C12A	0.9 (5)	C24B—C20B—C21B—C22B	1.8 (5)
C1A—C8A—C13A—C12A	−176.4 (3)	C19B—C20B—C21B—C22B	−108.7 (4)
C11A—C12A—C13A—C8A	−0.3 (6)	C24B—C20B—C21B—C23B	−141.4 (4)
C9B—C8B—C13B—C12B	−0.3 (5)	C19B—C20B—C21B—C23B	108.1 (4)
C1B—C8B—C13B—C12B	178.9 (3)	C25C—O3C—C24C—O2C	1.5 (5)
C11B—C12B—C13B—C8B	0.1 (6)	C25C—O3C—C24C—C20C	178.8 (3)
C18A—N1A—C14A—C15A	145.6 (3)	C19C—C20C—C24C—O2C	51.1 (6)
C17A—N1A—C14A—C15A	15.8 (3)	C21C—C20C—C24C—O2C	−18.4 (6)
C18A—N1A—C14A—C1A	−93.1 (3)	C19C—C20C—C24C—O3C	−125.9 (3)
C17A—N1A—C14A—C1A	137.1 (3)	C21C—C20C—C24C—O3C	164.6 (3)
O1A—C1A—C14A—N1A	−42.5 (3)	C25A—O3A—C24A—O2A	1.5 (6)
C8A—C1A—C14A—N1A	−158.8 (2)	C25A—O3A—C24A—C20A	178.6 (4)
C2A—C1A—C14A—N1A	77.6 (3)	C21A—C20A—C24A—O2A	−37.4 (6)
O1A—C1A—C14A—C15A	73.1 (3)	C19A—C20A—C24A—O2A	33.1 (6)
C8A—C1A—C14A—C15A	−43.2 (3)	C21A—C20A—C24A—O3A	145.6 (3)
C2A—C1A—C14A—C15A	−166.7 (2)	C19A—C20A—C24A—O3A	−143.8 (3)
C17C—N1C—C14C—C15C	13.7 (4)	C25B—O3B—C24B—O2B	−0.6 (7)
C18C—N1C—C14C—C15C	144.0 (3)	C25B—O3B—C24B—C20B	179.2 (4)
C17C—N1C—C14C—C1C	134.5 (3)	C21B—C20B—C24B—O2B	−34.9 (6)
C18C—N1C—C14C—C1C	−95.1 (3)	C19B—C20B—C24B—O2B	36.3 (6)
O1C—C1C—C14C—N1C	−48.6 (3)	C21B—C20B—C24B—O3B	145.3 (3)
C8C—C1C—C14C—N1C	−165.7 (2)	C19B—C20B—C24B—O3B	−143.4 (4)
C2C—C1C—C14C—N1C	70.9 (3)		

### Hydrogen-bond geometry (Å, °)

**Table:**

*D*—H···*A*	*D*—H	H···*A*	*D*···*A*	*D*—H···*A*
O1B—H1BA···N1B	0.86	2.08	2.682 (4)	127
O1A—H1A···N1A	0.84	2.30	2.650 (4)	105
O1C—H1C···N1C	0.84	2.31	2.679 (4)	107
C14C—H14B···O1B^i^	1.00	2.51	3.319 (4)	138
C19B—H19A···O2C^ii^	1.00	2.37	3.321 (4)	158
O1A—H1A···O2B^iii^	0.84	2.55	3.236 (4)	140

Symmetry codes: (i) *x*, *y*, *z*−1; (ii) *x*, *y*, *z*+1; (iii) −*x*+3/2, *y*−1/2, −*z*+1.
